# Challenges in the provision of healthcare services for migrants: a systematic review through providers’ lens

**DOI:** 10.1186/s12913-015-1065-z

**Published:** 2015-09-17

**Authors:** Rapeepong Suphanchaimat, Kanang Kantamaturapoj, Weerasak Putthasri, Phusit Prakongsai

**Affiliations:** 1 International Health Policy Program (IHPP), Ministry of Public Health of Thailand, Tiwanon road, Nonthaburi, 11000 Thailand; 2 Banphai Hospital, Banphai district, Khon Kaen, 40110 Thailand; 3 Department of Social Sciences, Faculty of Social Sciences and Humanities, Mahidol University, Nakhon Pathom, 73170 Thailand

**Keywords:** Providers, Health personnel, Migrants, Attitudes, Professional practices, Health services

## Abstract

**Background:**

In recent years, cross-border migration has gained significant attention in high-level policy dialogues in numerous countries. While there exists some literature describing the health status of migrants, and exploring migrants’ perceptions of service utilisation in receiving countries, there is still little evidence that examines the issue of health services for migrants through the lens of providers. This study therefore aims to systematically review the latest literature, which investigated perceptions and attitudes of healthcare providers in managing care for migrants, as well as examining the challenges and barriers faced in their practices.

**Methods:**

A systematic review was performed by gathering evidence from three main online databases: Medline, Embase and Scopus, plus a purposive search from the World Health Organization’s website and grey literature sources. The articles, published in English since 2000, were reviewed according to the following topics: (1) how healthcare providers interacted with individual migrant patients, (2) how workplace factors shaped services for migrants, and (3) how the external environment, specifically laws and professional norms influenced their practices. Key message of the articles were analysed by thematic analysis.

**Results:**

Thirty seven articles were recruited for the final review. Key findings of the selected articles were synthesised and presented in the data extraction form. Quality of retrieved articles varied substantially. Almost all the selected articles had congruent findings regarding language andcultural challenges, and a lack of knowledge of a host country's health system amongst migrant patients. Most respondents expressed concerns over in-house constraints resulting from heavy workloads and the inadequacy of human resources. Professional norms strongly influenced the behaviours and attitudes of healthcare providers despite conflicting with laws that limited right to health services access for illegal migrants.

**Discussion:**

The perceptions, attitudes and practices of practitioners in the provision of healthcare services for migrants were mainly influenced by: (1) diverse cultural beliefs and language differences, (2) limited institutional capacity, in terms of time and/or resource constraints, (3) the contradiction between professional ethics and laws that limited migrants’ right to health care. Nevertheless, healthcare providers addressedsuch problems by partially ignoring the immigrants’precarious legal status, and using numerous tactics, including seeking help from civil society groups, to support their clinical practice.

**Conclusion:**

It was evident that healthcare providers faced several challenges in managing care for migrants, which included not only language and cultural barriers, but also resource constraints within their workplaces, and disharmony between the law and their professional norms. Further studies, which explore health care management for migrants in countries with different health insurance models, are recommended.

**Electronic supplementary material:**

The online version of this article (doi:10.1186/s12913-015-1065-z) contains supplementary material, which is available to authorized users.

## Background

In recent years, cross-border migration has gained significant attention in high-level policy dialogues in numerous countries. According to the World Migration Report launched by the International Organization for Migration (IOM), the estimated total number of international migrants has reached 214 million, constituting over 3 % of the global population [1]. Between 1960 and 2005 there was an approximately two and a half fold increase in the number of people migrating across international borders, from 75 million to almost 191 million [2]. The Americas (North, Central, and South America, and the Caribbean) are the largest destination of international migrants, here alone the figures rose from 47 million in 2000 to more than 57.5 million in 2012 [3]. The same phenomenon was also found in Europe which has seen a consistent rise in the trend of migration since 2005, with migrants now constituting 8.7 % of the total European population [3]. It is noteworthy that 7–13 % of the foreign residents in Europe did not have a legitimate residence permit; as a result they were often labelled as ‘undocumented migrants’ [4, 5].

The growing trend of migration has been mirrored by a demand for a reorientation of health policies to better protect migrants’ health [6]. This fact is reflected by the content of a number of recent high-level, health-related international activities/meetings; In 2006, the United Nations General Assembly (UNGA) Global Commission on International Migration and the high-level dialogue called for a more collaborative and cohesive global response to the challenges of migration; In 2009, the Program Coordination Board (PCB) of the Joint United Nations Programme on HIV/AIDS (UNAIDS) held its 24th meeting in Geneva, highlighting HIV-related needs for people on the move. The Board also articulated that the improvement of HIV information and services for migrants would buttress the development and implementation of international healthcare strategies [7]. The issue of the health of migrants has expanded from disease-specific care to health promotion and disease prevention. For example, the annual European Public Health Association (EUPHA) Conference in 2014 underlined the need for adaptation of health promotion and disease prevention interventions for migrants and ethnic minority populations [8].

In addition, the World Health Organization (WHO) has been acting as a catalyst between various stakeholders, in addressing the health of migrants. Its action is visible through a number of relevant World Health Assembly Resolutions (WHR), for instance, WHR60.26 on ‘Workers health, global plan of action’, urging member states to work towards full coverage of all workers including migrants [9], and WHR61.17 on the ‘Health of migrants’, which called for migrant sensitive health policies and practices [10].

Challenges concerning the health of migrants cannot be tackled straightforwardly since the issue is highly dynamic and complicated, involving various stages of migration, from pre-departure to early and late migratory status [11, 12]. Furthermore, this matter is tightly intertwined with several social determinants, which are related not only to migrants’ characteristics (such as, different gender roles, cultural diversity, migration experiences, and precarious legal status), but also the contextual environment of migrant destination countries (such as, idiosyncratic health systems and cultural values) [13, 14].

Though there exists some literature exploring the health status and perception migrants have towards service utilisation in many receiving countries [15–17], there is still little evidence that deeply examines the health services migrants receive in actual practice from the viewpoint of service providers. This study therefore aims to systematically review the literature which has investigated the perceptions and practices of healthcare providers in managing care for migrants, as well as the challenges and barriers that health personnel faced.

## Methods

This study identified the following operating definitions. The review defined ‘healthcare providers/workers’ as people engaging in service delivery (in either the public or the private sectors) in structured healthcare facilities such as hospitals, primary care units, and community clinics. The definition was adapted from the WHO in 2006, which defined ‘health workers’ as ‘all people primarily engaged in actions with the primary intent of enhancing health’ [18]. However, in this review, family carers at home and health volunteers were excluded.

For service users, the main focus of this review was cross-country or international migrants, who had been residing in a destination country for a prolonged period, regardless of their legal status. Therefore asylum seekers and refugees were included whereas domestic migrants, foreign tourists, and transit visitors were excluded from the review. In should be noted that amongst migrants with precarious legal statuses there are subtle differences between the terms, ‘illegal migrants’, ‘undocumented migrants’, ‘irregular migrants’, etc. The operational definition of several subtypes of ‘illegal migrants’ is set out in Table 1. However these terms are often used interchangeably.

**Table 1:** Operational definition of ‘illegal migrant(s)’ applied for the review

Type	Definition
Irregular migrants	Irregular migrants are persons whose paths of migration did not adhere to legal provisions of entry and residence.
Undocumented migrants	Undocumented migrants are third-country nationals without a valid residence permit or visa allowing them to reside in the country of destination and who, if detected, may be liable to deportation.
Involuntary migrants	Any foreign-born people who have migrated to a country because they have been displaced from their home country, have an established or well-founded fear of persecution, or have been moved by deception or coercion.
Refugees	Any persons who have fled their country, are unable or unwilling to avail themselves of the protection of their country of nationality or habitual residence because of a well-founded fear of persecution on account of race, religion, nationality, membership of a particular social group or political opinion.
Asylum seekers	Asylees are persons being granted asylum, having the right to remain permanently in destination country. Contrasting to a refugee who underwent processing overseas, an asylee is a person who first reached another country, usually as a visitor or other non-immigrant status, and either upon or after arrival declared oneself to be a ‘refugee’ based on the refugee standard.

Source: adapted from

1. Biswas et al. (2001) [5]

2. Walker and Barnett (2007) [92]

### Conceptual framework

**Fig. 1:**
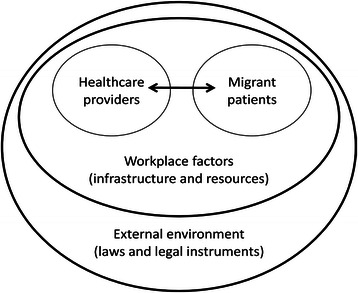
Conceptual framework of the systematic review. Source: adapted from the ‘Four-Level Model of Health Care System’ by Ferlie and Shortell [19]

The conceptual framework for this review was adapted from the ‘Four-Level Model of Health Care System’ by Ferlie and Shortell [19], see Fig. 1. The model suggested that the success of health care delivery was dependent on the performance and integration of the health system at different levels, namely: (1) individual patient level, (2) care team level (e.g., clinicians, pharmacists, and others), (3) organisation or workplace level (e.g., hospital, clinic, nursing home, etc.), including infrastructure and complementary resources, and (4) societal level (e.g., legal framework, cultural value, and country economics). This model was selected based on the hypothesis that challenges faced by a provider were shaped not only by individual attitudes towards migrant clients, but also by surrounding constraints where s/he was operating. Consequently, the review findings were analysed from the following angles: (1) interaction between healthcare providers and migrant patients, (2) interaction between healthcare providers and their workplace context, and (3) influence of other external factors, specifically laws and regulations that stipulated the right to health care of migrant clients.

### Search strategy

This review sought a broad description of the attitudes and perceptions of healthcare providers, as well as the challenges they faced in the provision of services to cross-border migrants; articles which utilised qualitative methods were considered more suitable in achieving this objective than articles using quantitative methods. Accordingly, the search terms were applied in search of qualitative research articles.

Potential articles were recruited from two main strands: (1) systematic search, and (2) purposive search. In the systematic search, articles in areas of medicine, nursing and psychology, and health science were examined. Three key search engines, namely, Medline, Embase and Scopus, were explored. In Medline, both ‘Medical Subject Headings (MESH)’ search, and text search were executed. In Embase and Scopus, where MESH terms are not available, an exploding search strategy was applied in order to encompass relevant texts as though the MESH terms were explored. The search was done in the keywords, abstracts and titles of articles. The publication date was limited: only studies conducted between 1^st^January 2000 and 30^th^June 2015 were included. Due to limited capacity in language translation, studies published in any language other than English were excluded. Most articles were retrieved from the library of the International Health Policy Programme, the Ministry of Public Health, Thailand. Articles unavailable at the library were recruited from other network libraries. Table 2 indicates the search terms employed in each database. Truncation and wildcards were carefully checked in all search engines.

**Table 2:** Search terms used in Medline, Embase, and Scopus for the systematic review

Search engine	Search terms
Medline	(((((“mixed method*”)) OR ((“qualitative study”)) OR ((“qualitative research”)) OR ((“Qualitative Research”[Mesh])))) AND ((((“Transients and Migrants”[Mesh])) OR ((“Emigrants and Immigrants”[Mesh])) OR ((“migrants”)) OR ((“refugees*”)) OR ((“asylum seekers*”))) AND (((“Health Services”[Mesh])) OR ((“Professional Practice”[Mesh])) OR ((“Attitude of Health Personnel”[Mesh]))) AND ((“Health Personnel”[Mesh]))))
Embase	(((exp migrant/) OR (exp refugee/) OR (exp asylum seeker)) AND (exp health care personnel/) AND ((exp health personnel attitude/) OR (exp professional practice/) OR (exp health service/)) AND ((exp qualitative research/) AND (“mixed method”.mp.)))
Scopus	(TITLE-ABS-KEY (“qualitative research” OR “qualitative study” OR “mixed method”) AND PUBYEAR > 1999 AND PUBYEAR < 2016) AND (((((TITLE-ABS-KEY (“asylum seekers”) AND PUBYEAR > 1999 AND PUBYEAR < 2016) OR (TITLE-ABS-KEY (refugee) AND PUBYEAR > 1999 AND PUBYEAR < 2016) OR (TITLE-ABS-KEY (immigrant) AND PUBYEAR > 1999 AND PUBYEAR < 2016) OR (TITLE-ABS-KEY (migrant) AND PUBYEAR > 1999 AND PUBYEAR < 2016)) AND ((TITLE-ABS-KEY (“health personnel”) AND PUBYEAR > 1999 AND PUBYEAR < 2016))) AND (((TITLE-ABS-KEY (“health service”) AND PUBYEAR > 1999 AND PUBYEAR < 2016) OR (TITLE-ABS-KEY (“attitude”) AND PUBYEAR > 1999 AND PUBYEAR < 2016) OR (TITLE-ABS-KEY (“practice”) AND PUBYEAR > 1999 AND PUBYEAR < 2016))))) AND (LIMIT-TO (LANGUAGE, “English”))

Source: Authors’ synthesis

For the purposive search, articles and publications were retrieved from the WHO website (http://www.who.int/hac/techguidance/health_of_migrants/en/) and the freely-accessed online grey literature database organised by the New York Academy of Medicine Library (http://www.greylit.org/library/search#wt=json&facet=true&q=migrant&qt=dismax&fl=id&qf=full_text&facet.field=publisher&facet.field=full_subjects&q.op=AND&start=0).

### Inclusion and exclusion of articles and data extraction

Abstracts of the initially selected studies were independently screened by two reviewers (RS and KK). Given any disagreement between the reviewers, an internal meeting would be held until a consensus was reached. The articles which passed the screening process would be read in full, and analysed for the key message by all co-authors. Eligible studies were included when they met all the following criteria: (1) providing information about perceptions, attitudes or practices of healthcare providers, (2) presenting evidence relevant to cross-country migrants regardless of their legitimacy of residence permit, (3) involving healthcare services that were commonly performed in clinical services in a real world setting, in either the public or private sector, and (4) being primary research with scientific details of the research aims and methods used.

Articles were excluded from the review if they met any one of the following criteria: (1) failing to provide sufficient information about providers’ perceptions, attitudes, practices; (2) engaging with domestic migrants, or members of the indigenous population, rather than cross-country migrants, (3) not employing a rigorously scientific approach (that is, a selected article must pass the first two questions of the quality assessment checklist; more details are presented below in the ‘Quality assessment and data analysis’ subsection) or just representing an author’s opinion, that meant that letters to the editor or commentary articles were left out, (4) not relevant to western or widely practiced modern medicine (thus, health services, which were very specific to some cultures, such as Aruyaveda or Chinese herbal medicine, were excluded), and (5) were restricted to experimental or biomedical pilot programmes (e.g., vaccine pilot programmes or clinical drug trials).

Potential articles were then checked for duplication and the full text was screened. Studies were stored and tracked in a manageable computerised form by EndNote software Version X4.

### Quality assessment and data analysis

The main findings of each selected article were extracted and collected in the data extraction form. A quality assessment tool was applied from Spencer et al. [20] and the CASP checklist [21]. The checklist had 10 questions, each of which would be given an answer, ‘Yes’, or ‘No’, or ‘Cannot tell’. Passing the first two screenings questions meant that an article’s research question clearly matched the review objective, and secondly, the methods used were appropriate in addressing the research question. In this case the article’s full text would then be retrieved and perused in greater detail. Articles which failed to meet the above screening criteria would not be presented in the data extraction table. For example, the study by Grewal [22] was excluded since it aimed to describe health beliefs of perinatal care amongst Indian women in Canada through users’ perspectives rather than through providers’ perspectives.

It should be noted that the quality assessment tool did not aim to apply a specific cut-off point to eliminate articles of seemingly poor quality. Instead it was used to remind audiences of any potential bias which might occur in a study. The analysis tool was applied from ‘Methods for the thematic analysis of qualitative research in systematic reviews’ by Thomas and Harden [23], which was composed of two steps. First, the extracted data were read and coded manually, then their meanings were captured and charted against the above framework, constructing so-called descriptive themes, which were reported in the results section. The second step was constructing higher-level themes (conceptual or analytical themes) from the descriptive themes [24].

## Results

**Fig. 2:**
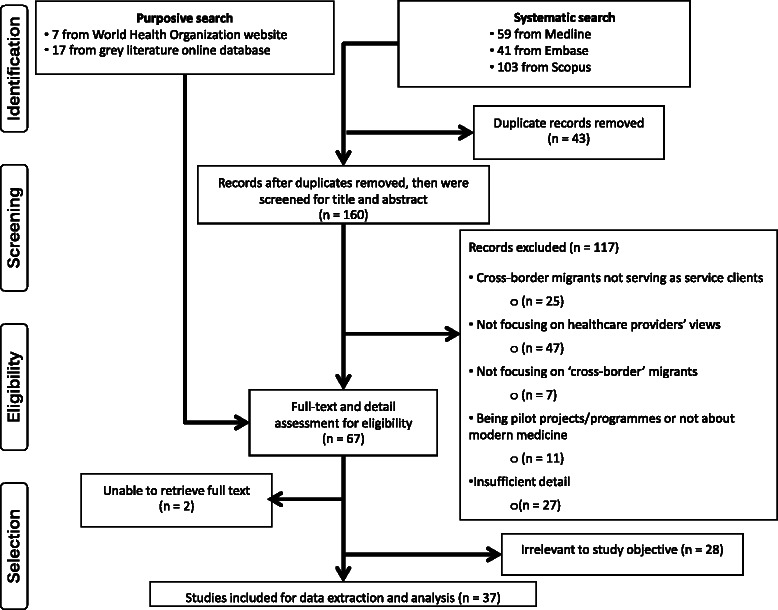
Article selecting process. Source: Authors’ synthesis

An overview of the article selection process is demonstrated in Fig. 2.

A total of 203 articles were retrieved from the systematic search (41 from Medline, 59 from Embase and 103 from Scopus). After dropping 43 duplicated articles, there were 160 remaining articles. In the purposive search, 17 articles from the WHO website and 7 articles from the grey literature database were initially selected. Thus, after combining articles from both search strands, there were 184 articles for abstract screening.

Of the 184 articles, 37 passed the screening process and then the full text was explored for key messages. The Kappa inter-rated agreement coefficient appeared to be 0.85 (*P*-value <0.001), suggesting a high level of agreement. The key finding of each study was extracted and synthesised together as presented in the Additional files 1 and 2, and the quality assessment result of each article is displayed in Table 3.

**Table 3:** Quality assessment of the selected articles

Selected articles (author(s), year)	Q1	Q2	Q3	Q4	Q5	Q6	Q7	Q8	Q9	Q10
1. Abbot and Riga (2007) [25]	Y	Y	Y	N	Y	Y	?	Y	?	Y
2. Akhavan (2012) [26]	Y	Y	N	Y	Y	Y	Y	Y	?	Y
3. Boerleider et al. (2014) [27]	Y	Y	Y	Y	Y	?	Y	Y	Y	Y
4. Briones-Vozmediano et al. (2014) [28]	Y	Y	?	Y	Y	N	Y	Y	Y	Y
5. Byrskog et al. (2015) [29]	Y	Y	Y	Y	N	Y	Y	Y	?	?
6. Cross and Bloomer (2010) [30]	Y	Y	N	?	Y	N	Y	Y	?	N
7. Dauvrin et al. (2012) [31]	Y	Y	?	?	Y	N	Y	Y	Y	Y
8. Donnelly and McKellin (2007) [32]	Y	Y	N	?	?	N	?	N	Y	?
9. Eklof et al. (2015) [33]	Y	Y	N	?	Y	N	Y	?	Y	Y
10. Englund and Rydstrom (2012) [34]	Y	Y	?	?	Y	N	Y	Y	Y	Y
11. Farley et al. (2014) [35]	Y	Y	?	?	Y	N	?	Y	N	?
12. Foley (2005) [36]	Y	Y	Y	?	Y	N	Y	?	Y	N
13. Fowler et al. (2005) [37]	Y	Y	Y	Y	Y	N	Y	?	?	Y
14. Goldabe and Okuyemi (2011) [38]	Y	Y	?	N	?	N	?	Y	?	Y
15. Hakonsen et al. (2014) [39]	Y	Y	Y	N	Y	N	Y	Y	Y	Y
16. Health Protection Agency (2010) [40]	Y	Y	?	?	?	N	Y	?	Y	?
17. Hoye and Severinsson (2008) [41]	Y	Y	Y	?	Y	N	Y	Y	?	Y
18. Hultsjo and Hjelm (2005) [42]	Y	Y	Y	?	Y	N	Y	?	Y	Y
19. Kurth et al. (2010) [43]	Y	Y	Y	Y	Y	N	?	?	Y	?
20. Lindsay et al. (2012) [44]	Y	Y	Y	Y	Y	N	Y	?	Y	Y
21. Lyberg et al. (2012) [45]	Y	Y	Y	?	Y	N	Y	Y	Y	Y
22. Manirankunda et al. (2012) [46]	Y	Y	Y	Y	Y	N	?	Y	Y	Y
23. Munro et al. (2013) [47]	Y	Y	Y	?	?	N	?	?	Y	?
24. Nicholas et al. (2014) [48]	Y	Y	Y	Y	Y	N	?	Y	Y	Y
25. O’mahony and Donnelly (2007) [49]	Y	Y	?	?	Y	N	Y	Y	Y	Y
26. Otero-Garcia et al. (2013) [50]	Y	Y	?	?	?	N	Y	Y	Y	?
27. Pergert et al. (2008) [51]	Y	Y	?	?	Y	Y	Y	?	Y	Y
28. Rosenberg et al. (2006) [52]	Y	Y	Y	Y	Y	?	Y	Y	?	Y
29. Samarasinghe et al. (2010) [53]	Y	Y	Y	Y	Y	Y	Y	Y	Y	?
30. Sandu et al. (2013) [54]	Y	Y	?	Y	Y	Y	Y	?	Y	Y
31. Straßmayr et al. (2012) [56]	Y	Y	Y	N	Y	N	?	Y	Y	Y
32. Suurmond et al. (2013) [55]	Y	Y	N	Y	Y	N	Y	Y	Y	?
33. Terraza-Nu’n˜ez et al. (2011) [57]	Y	Y	N	Y	Y	N	N	Y	Y	Y
34. van den Ameele et al. (2013) [58]	Y	Y	Y	Y	Y	N	?	Y	Y	Y
35. Vangen et al. (2004) [59]	Y	Y	?	Y	Y	N	Y	Y	Y	Y
36. Wachtler et al. (2006) [60]	Y	Y	N	?	?	?	?	?	Y	N
37. Worth et al. (2009) [61]	Y	Y	?	Y	Y	N	Y	?	Y	Y

Note

• Q1 = Was there a clear statement of the research aim?

• Q2 = Was a qualitative methodology appropriate?

• Q3 = Was the research design appropriate to address the research aim?

• Q4 = Was the recruitment strategy appropriate to the research aim?

• Q5 = Was the data collected in a way that addressed the research issue?

• Q6 = Was the relationship between researcher and participants sufficiently considered?

• Q7 = Were ethical issues taken into consideration?

• Q8 = Was the data analysis sufficiently rigorous?

• Q9 = Does the research have a clear statement of the findings?

• Q10 = Does the report sufficiently express the research value?

• Y = Yes (clearly described)

• N = No (Not described)

• ? = Cannot tell (described but with limited detail)

Of the 37 articles [25–61] three are presented data from multi-country surveys [31, 54, 56], the remaining thirty-four were standalone study projects [25–30, 32–53, 55, 57–61]. About 68 % of the reviewed studies (25/37) took place in Europe, followed by 24 % (9/37) in America, 5 % (2/37) in Australia and 3 % (1/37) in Africa. Only two studies were carried out in developing nations: one in America (Costa Rica) and the other in North Africa (Morocco) [38, 58].

The quality appraisal found that the quality of the selected articles varied substantially. Though all the selected articles clearly specified the research question and the methodology used, only some articles had justified why such research designs were employed, for instance, Hoye and Severinsson [41] and Hakosen et al. [39]. The most common quality issue was the failure to consider the issue of reflexivity and to critically examine the extent of potential bias, or influence on the findings, resulting from the role and experience of the researchers. Examples of articles which had a clear message acknowledging the reflexivity matter, were Abbot and Riga [25], Akhavan [26] and Byrskog et al. [29].

According to the review framework, the key messages from the review were presented as the following.

### Interaction with immigrant patients

Almost all the selected articles had congruent findings regarding language and cultural challenges, and a lack of knowledge of a host country’s health system amongst many migrant patients [25, 39, 50, 51, 55, 57]. Such difficulties critically impeded effective communication between migrants and providers. The barriers interweaved with specific cultural beliefs, such as patriarchal norms in Muslim culture, which meant that healthcare providers were incapable of addressing migrants’ illnesses in a holistic fashion. In light of this difficulty, healthcare staff, particularly primary care physicians, were reluctant to delve into details beyond ‘physical’ illness. Consequently, they shaped their practice to be more ‘superficial’ and ‘straight forward’. This meant that ‘hidden’ illnesses such as stress or other mental health disorders, which may have been caused by traumatic experiences in immigrants’ country of origin, bereavement or by facing terminal disease, were left unresolved. This problem was highlighted by the study of Rosenberg et al. [52] and Hultsjo and Hjelm [42]: they found that language barriers made nurses in psychiatric emergency wards adapt the way they took patients’ medical histories to be less complex and to avoid delving into the traumatic experiences of migrants in their countries of origin.

Dauvrin et al. [31] reported that providers in accident and emergency (A&E) departments, where treatment was more direct, were far less affected by language and cultural divergence than those in mental health and primary health care clinics, where care was expected to be more holistic. Samarasinghe et al. [53] described that about one fifth of the primary health care nurses (PHCNs) in Sweden confined their service to purely somatic diseases, despite the fact that family problems or mental illnesses had not been properly disentangled. A similar finding was identified in a case of sexual violence described by van den Ameele et al. [58].

Furthermore, cultural beliefs, specifically gender preferences, also played an important role. This influence contributed to difficulties in service provision. As expounded by Lyberg et al. [45], male interpreters often did not understand the needs of immigrant women receiving maternity care. Hoye and Severinsson [41], and Englund and Rydstorm [34] reported that female nurses often perceived a lack of respect from some immigrant patients. Where health services could not be delivered effectively, health professionals occasionally perceived mistrust from their immigrant patients. This situation had made providers fear accusations of racism if they unintentionally made cultural mistakes in their clinical practice [46, 61]. Moreover, the problem of mistrust of health professionals was more complicated by migrant patients lack of familiarity with the health system [34, 49, 54].

### Interaction with providers’ workplaces/organisations

Most respondents expressed concerns over in-house constraints resulting from heavy workloads and the inadequacy of human resources and institutional capacity [26, 42, 56]. As discussed by Strabmayr et al. [56], such challenges were more apparent when providers with highly-specific expertise were in-demand, for example, the shortage of skilled psychotherapy health personnel in mental-health care units in Europe.

To address communication challenges, interpreting services were set up, providing either face-to-face or telephone assistance. Nevertheless, the availability of interpreting services neither guaranteed the quality of care, nor did it ensure the interpreting service would in practice be utilised. Akhavan [26] and Farley et al. [35] underlined that although healthcare facilities recognised the importance of having interpreting services, using interpreters was somewhat time consuming. Eklof et al. [33] and Lindsay et al. [44] emphasised that using phone interpreters increased the workload of nursing staff, especially in situations requiring urgent care. Besides, Lyberg et al. [45] found that in some circumstances, such as, during delivery and maternity care, an interpreter service was of little use.

The lack of quality interpreters was not the only problem. A lack of diversity in the ethnic backgrounds of healthcare staff was considered another key hurdle in the provision of cross-cultural care. Nicholas et al. [48] mentioned that a key solution to solve this problem is finding healthcare staff who were able to serve as ‘cultural brokers’, bridging between the needs of migrants and the understanding of healthcare providers.

Most respondents participating in the selected studies worked in a workplace where service provision guidelines at times contradicted, or at least did not match, the attitudes and beliefs of migrant patients [36, 37, 41, 47, 61]. Foley [36] provides an example where nurses in a US HIV clinic changed their routine practice by delivering medicine for HIV positive migrants at places outside the patients’ homes. This adaptation was made to avoid disclosing the HIV status of female migrants to their male partners. Hoye and Severinsson [41] underscored the fact that the mismatch betweenroutine clinical service guidelines and migrant patients’ beliefs had increased the stress of healthcare staff [60]. An instance of intensive care wards in Norway was raised to support this notion since these wards were often crowded by a large number of family members of immigrant patients, and this hampered the normal care procedures of the nursing staff [41]. Another example was raised by Vangen et al. [59], gynaecologistsin Norway felt great pressure when providing delivery care to pregnant African women who had experienced infibulation. Defibulation was not routinely performed under the existing workplace guidelines, consequently many professionals occasionally performed caesarian sections for these infibulated migrant women in lieu of defibulation. This adaptive practice might lead to unwanted clinical outcomes for both mothers and their newborns [59].

### Interaction with laws,professional standards, and the health system of the host country

Professional norms strongly influenced the behaviours and attitudes of healthcare providers. In cities where policy towards universal access was open for ‘everybody’ clinical practice was more relaxed. However, the relaxation of laws that granted illegal migrants the right to health care did not guarantee that migrants would be able to access care in real life. Administrative and financial burdens often played an important role in limiting the right to healthcare of migrants, particularly the undocumented ones [32, 33, 36, 47]. Donnelly and McKellin [32] showed a case in Canada where breast cancer screening services for immigrants faced the biggest funding cutbacks. Because of administrative delays, refugees and refugee claimants in Quebec found themselves uninsured despite having the right to participate in the Interim Federal Health Programme [47]. Similar challenges was also found in the US. In order to be insured at the city health centres in Philadelphia, immigrant patients must first provide proof of residence to the accountable authority. Yet, some African women often had no documentation in their own name because they lived with male partners or relatives [36].

By contrast, in countries where legal policy restricted access to healthcare for undocumented migrants most health practitioners did not feel obligated by these city mandates. As a result, informing the police or government authorities about the presence of illegal migrants was an uncommon practice, even though they were compelled, by law, to do so [31, 43, 58]. Common excuses used by healthcare providers were grounded on philanthropic concepts, they recognising migrants as a vulnerable population and tool into account the potential threat to the public of leaving sick migrants untreated [38].

Goldabe and Okuyemi [38] highlighted that in Costa Rica, undocumented migrants were barred by law from accessing public health services with only 3 exceptions, namely: emergency services, health care for children and adolescents under 18 years of age, and prenatal care. In the opinion of healthcare providers allowing access to these services seemed to be reasonable since it was beneficial in preventing the country from experiencing public health threats. Many providers, however, stated that healthcare benefits for undocumented migrants should not include treatment for occupational injuries because the profit from the treatment of these injuries did not benefit the health of the wider national population but instead benefitted individual companies [38].

Another key common finding from the review is that many healthcare providers utilised ‘informal networks’ to overcome administrative and referral barriers in managing services for illegal migrants. This might be due to the fact that non-government organisations (NGOs) or philanthropic agencies were less bound by rules and procedures than government authorities [40, 56]. Respondents in the UK briefly described confusion in National Health Service (NHS)’s regulations, which limited some benefits (e.g., housing aids) for certain types of migrants, such as vulnerable adults, migrants’ relatives and dependants. Some UK health professionals thus entrusted non-statutory organisations or civil networks to fill this service gap [40].

## Discussion

It is undeniable that services for migrants are dynamic and impacted not only by providers’ individual attitudes, but also by the health need of migrants and their family members, as well as the influence of underlying health system, legal implication and social values. Regarding the review findings, two conceptual themes were identified, namely: ‘Complexities in managing health care in a culturally-sensitive manner in light of resource constraints and the fear of making cultural mistakes’, and ‘Professional ethics in light of restrictive healthcare policy’. Note that relevant references, which failed to pass the screening process and did not appear in the data extraction table, might also be discussed in the following section in order to support or contest the review findings.

### Complexities in managing health care in a culturally-sensitive manner in light of resource constraints and the fear of making cultural mistakes

Theoretically, culturally-sensitive health care is perceived as an effective means for promoting better health statuses for immigrants [62, 63]. This can help overcome the powerlessness that immigrants often feel when they are excluded from the dominant culture of their destination countries. However, applying the concept of cultural sensitivity into real practice is not straightforward. The problems of using an interpreting service from the review above provide an obvious example. Akhavan [26] and Farley et al. [35] suggested that while healthcare providers recognised the merit of using an interpreting service, the practice of using interpreters was labour intensive and time consuming. These challenges presented in a context in which healthcare providers already faced a shortage of financial and human resources. The congruent findings reported by Eklof et al. [33] and Lindsay et al. [44], demonstrated that using phone interpretation services significantly increased the workload of nursing staff. Bischoff and Hudelson [64] provided additional evidence which showed that, although, using ‘professional interpreters’ was considered as ‘gold standard’ for providing multi-cultural care, hiring bilingual interpreters did not guarantee high quality culturally-sensitive care. Specifically, problems arose when the interpreters were able to overcome the ‘language’ difficulties but still lacked a clear understanding of migrants’ behaviours and beliefs [64, 65]. Binder et al. [66] and Lyberg et al. [45] found that in some circumstances, such as, during delivery and maternity care, interpretation services were of little use. In summary, the complexity of overcoming language barriers could not be solved solely by ‘hiring’ interpreters; successfully overcoming these barriers also required identifying whether or not the service matched needs of migrant beneficiaries.

While many studies recommended the use of professional interpreters, there is still room for using family interpreters. Although the use of family interpreters was not considered standard clinical practice physicians still accepted this in some situations, specifically when clinical presentations were uncomplicated (e.g., cough, cold, fever, etc.). Gray et al. [67] suggested that refugees and migrants with limited English proficiency (LEP) in New Zealand preferred relying on their bilingual relatives to using professional interpreters. In contrast to Australia, where telephone interpreter was freely accessible, healthcare providers in New Zealand had to shoulder the cost of a landline interpreting service; this resulted in a low utilisation rate of telephone interpretation support [67]. Some providers avoided face-to-face interpretation by preparing translated materials, like leaflets or videotapes, which were especially useful in maternal care. Yet, the efficacy and effectiveness of using those materials had not been explored [45].

Another noteworthy issue was identifying how to deliver healthcare services in a ‘culturally-sensitive’ manner without creating a sense of ‘discrimination’ or ‘racism’. An obvious instance was depicted by Manirankunda et al. [46] in Flanders, Belgium. The study was centred on provider-initiated HIV testing and counselling (PITC) for Sub-Saharan African migrants (SAMs). Though PITC was deliberately initiated to tackle high prevalence of HIV/AIDS amongst the SAMs population, physicians were reluctant to encourage their patients to undertake HIV/AIDS testing (unless patients themselves requested) owing to a fear of being accused of racism. A survey of the opinions of health experts in Greece failed to reach a consensus on whether or not establishing a separate ward for migrants would increase the efficiency of healthcare service delivery, this resulted from fears that it might emphasise the perception of racial inequity [68]. This is something Worth et al. [61] called the ‘fear of making cultural blunder’ in health practitioners. In occupational health, even though racism is not expressed explicitly, Meershoek et al. [69] observed that Dutch doctors assigned a stereotype of ‘problematic’ patients to migrants more often than to Dutch patients. This bias explained why Dutch occupational physicians occasionally failed to fine tune their coaching activities to meet the needs of migrants, and this, in turn, made some migrants more likely to suffer from conditions that prevented them from working than general Dutch patients.

A similar issue was found in debates on tuberculosis screening of asylum seekers in the UK; is it a protective measure for the benefit of all UK residents, or another kind of racial discrimination [70]? Bracanovic [71] argued that being ‘culturally sensitive’ in bioethics was implausible for the following reasons: (1) it rendered the disciplinary boundaries too flexible and was inconsistent with Western biomedical sciences, (2) it was practically useless because it approached cultural phenomena in a predominantly descriptive and selective manner, and (3) it indirectly justified certain types of ‘discrimination’. Swendon and Windsor [72] also mentioned that modern-day misunderstandings of multiculturalism in health care policy tended to perpetuate beliefs of ‘racial superiority’.

### Professional ethics in light of restrictive healthcare policy

Managing services for undocumented migrants is affected by the laws and regulations in which a health facility is operating [73]. It is clear that almost all international law and legal instruments have (theoretically) secured migrants human rights, including their right to health care [74, 75]. However, there are diverse ways of interpreting the law when it comes to real practice. As a consequence, substantial country-to-country and within-country variations were observed. These variations concerned the types of migrants permitted to be insured, the type and range of services, and differing levels of financial protection [73].

Dauvrin et al. [31] displayed the variation in legal provisions for undocumented migrants in16 European countries according to the level of care by categorising the surveyed countries into 3 subsets, namely, (1) countries allowing migrants to enjoy (almost) the full range of care (eg, France, Italy, Spain, etc.), (2) countries only allowing access to emergency services and certain primary care services (eg, Austria, Belgium, Denmark, etc.) and (3) countries denying the right to access health services at almost all levels and types of care (eg, Finland and Sweden). Marrow [76] raised a distinct case in San Francisco where illegal migrants were ‘semi-legalised’ by the recognition of their residence permit in the city, thereby, rights to care were endorsed to a larger extent in San Francisco than elsewhere in California or in many other states in the US.It should be noted that the legal instruments, which ratified migrant’s right to care, cannot be exercised perpetually; they have been influenced by, and have shifted with, political and economic changes. This phenomenon oftentimes led to bewilderment amongst healthcare management as some health practitioners have been unable to keep pace with the rapid changes. An intense debate was heard in 2012 in Spain when the government made a substantial change in the national health care system. This limited the right of non-Spanish inhabitants, who lacked legitimate residence permits, to access health services (except for maternal and emergency services). The reform was backed by the Court of Auditors which argued that insured Spanish citizens were bearing high healthcare costs incurred by non-Spanish citizens; thus, the reform was justifiable [77].

Aside from the framework of civil law and regulation, the practices of healthcare providers were constructed under health professional norms and ethics, the primary intention of which is to secure the health interest of all human beings regardless of ethnicity or nationality [47, 78].

It seems that ‘formal’ health professionals (eg., physicians and nurses) still have ‘margins’ or ‘loopholes’ which enable them to exercise their discretion in protecting the interests of patients, even though, to some extent, such practices contradict the law [79]. Priebe et al. [80] explained several strategies/tactics whereby physicians, who worked in cities where migrants’ right to care was restricted, circumnavigated the obstacles of limited entitlement to health benefits and avoided unwanted financial burdens on migrants. These tactics included referring their clients to charitable NGOs or ordering laboratory samples in the physicians’ name. Strabmayr et al. [56] labelled such adaptive behaviour as ‘turning a blind eye’. Reporting the presence of illegal migrants to the police was undertaken only in special circumstances, such as when migrants were considering getting involving with crime or when they had risky behaviours which might pose a threat to the public [58, 81].

In contrast to health professionals, supporting staff seemed to use those tactics less than health practitioners since the non-clinical staff were less bound by professional norms [82]. Hargreaves et al. [83] also observed that NHS payment officers in the UK, who were accountable for medical expense claims, had played a critical role in determining whether or not the overseas visitors were eligible to be exempted from charges for primary care service.

Vanthuyne et al. [78] attempted to gain a deeper understanding of how providers strike a balance when torn between ‘professional ethics’ and ‘legal responsibilities’. Those arguing against universal access perceived illegal migrants to be abusing the host country’s health system and even expropriating resources (which were always sparse) from the native population; while on the other end of the continuum, some health professionals perceived uninsured migrants to be ‘deserving of free care’ on the basis of ‘right’. Interestingly, some respondents in that study found a compromise by designating migrants with precarious legal statuses as ‘vulnerable’ groups, whose ‘right to care’ became a ‘privilege’; thus care was given based on a principle of humanitarian aid or philanthropy, rather than as a ‘right’ [78].

Interestingly, stricter and more complex rules governing the normalisation of migrants’ immigration status have not discouraged the influx of migrants. Though this was not specifically identified in any of the selected articles, this was demonstrated in some international publications. Having analysed the immigration history of Mexicans in the US, De Genova [84] suggested that even in a period when immigration law became ostensibly stricter, it did not deter migration but rather generated a shift from legal to illegal migration. Van Der Leun [85] pointed out that the Linking Act in the Netherlands, which aimed at excluding illegal migrants from using public services, caused an obvious tension on the local-level staff due to the way it shifted the responsibility for limiting migrants rights to care from immigration control officers at the country border, to local healthcare staff. The United Kingdom Trade and Investment Department suggested that the recent tightening of immigration laws in the UK would worsen the country’s current economic recession, and also increase the unemployment rate [86].

The contradiction between the law and professional ethics might create tension and misunderstandings between healthcare providers and their migrant patients. Lyons et al. [87] suggested that poor relationships between providers and patients might contribute to adverse effects on public health as a whole because migrants would be likely to sneak out from regular/formal health services, and therefore remain untreated. A relevant finding was presented by Biswas et al. [5] who described how doctors in emergency wards in Denmark perceived a sense of mistrust amongst South Asian undocumented migrants, this was evidenced by the use of ‘false identification’ (using another person’s name when visiting a facility instead of using their real name) by some migrants when utilising services.

### Strengths, weaknesses and limitations

This study has a key strength in gathering cutting edge evidence about how providers perceived, and adapted themselves in delivering care to migrants in their daily practice. However, despite a rigorously designed method, the review still had some weaknesses and limitations.

The first methodological limitation was that the search strategy did not encompass non-English-language articles due to limited interpreting capacity. Secondly, the majority of articles were retrieved from online databases and their selection was largely based on the MESH search strategy. Despite recruiting some grey literature from key international agencies, the grey literature from other sources, such as university-based reports, and unpublished articles and domestic text books, were likely to be left behind. This point is very important since migrants’ health is very context-specific. Individual country reports might have explored this topic more deeply than peer-reviewed publications.

Lastly, quality assessment was not executed in an enumerating/scoring system, which is conventionally done in most systematic reviews and meta-analysis. The reason for not using a quality assessment score stemmed from the fact that, since this review aimed to capture a broad understanding of the perceptions, attitudes and practices of healthcare workers providing services for immigrants, having a great miscellany of evidence (sensitivity) was deemed preferable than recruiting only studies with good quality (specificity). This is the so-called ‘configuring’ approach as described by Gough et al. [88] and Voils et al. [89]. The configuring approach is a method for synthesising research in which findings are used to explain and modify theoretical or narrative renderings of the target outcomes. Unlike configuration, ‘assimilation’ is an approach in which findings are pooled together in order to answer a specific research question [88, 89]. Atkins et al. [90] suggested that appraising the quality of qualitative studies might be an exercise in judging the quality of the written report rather than the research procedure per se. Articles published in qualitative-oriented journals were easier to evaluate since the length of articles allowed the authors to give details on the research process. Thus, evaluating the relative merits of the articles was not the primary concern in this case. In contrast, the quality reporting here aimed to remind the audience about the limitations of each study should its findings be applied in a real life setting.

Regarding limitations in the study results, firstly, it is important to remember that most of the selected studies were produced in developed countries in Europe; only two articles were from developing nations [38, 58]. This issue might limit the generalisation of the review findings. It should be noted that there was no distinct difference in the study results between countries with differing economic statuses, or between countries with different health insurance systems. However, each country has introduced different rules and regulations in guaranteeing migrants’ right to health care (for example, the UK health system allows undocumented migrants to utilise emergency care, primary care, and treatment for some infectious diseases; in Switzerland, undocumented migrants must buy private health insurance under public supervision in the same way as Swiss citizens) [91]. Future studies that deeply explore and collate evidence from countries with different types of health insurance models were recommended.

Secondly, the legal/citizenship status of migrants is very dynamic. Migrants with secured legal status may become illegal migrants if they stay in a host country longer than the visa permission; and, on the other hand, the status of undocumented migrants may be legalised once they register themselves with the state authorities. Most of the articles presented in this review focused on health services for migrants with precarious legal status, such as refugees, irregular migrants and undocumented persons. The review hardly explored the status of more affluent migrants, for instance, tourists, expatriates, and foreign businessmen. Accordingly, generalisations of the study’s findings to other types of migrants should be made with caution.

Thirdly, not all aspects of providers’ attitudes were explored. The review reported much about how healthcare providers addressed language barriers and contradictions between professional norms and the law, however, the measures taken to overcome challenges caused by different cultural and religious beliefs were sparsely reported.

Lastly, the reported perceptions and practices of healthcare providers demonstrated in this review were mainly drawn from the subjective assessment of the participants. Almost all of the articles employed in-depth interviews and focus group discussions as their primary data collection tools. Hence, it is possible that the reported perception of the respondents might be different from their real clinical practice. Further studies, which devise a variety of data collection techniques (such as observations, document studies, etc.) will be of great benefit in the development of appropriate healthcare system for migrants in the future.

## Conclusion

Given the great variation in the provision of healthcare services for migrants, the review found that the perceptions, attitudes and practices of individual practitioners providing services for migrants were markedly influenced by several factors. Diverse cultural beliefs and language differences made it more difficult for service providers to meet the needs of migrants, and these problems could not be addressed by merely establishing an interpreting assistance. Limited institutional capacity, either in terms of time or resource constraints, as well as a fear of perceived racism, had rendered the provision of culturally-sensitive care more complex. Professional ethics, which aimed to protect the interests of patients, often contradicted legal mandates that tended to restrict the right to health of migrants. Nevertheless, practitioners attempted to address this problem by partially ignoring the immigrants’ precarious legal status, and used various tactics to keep their clinical practice functioning in accordance with their professional norms. Further studies, which explore migrant healthcare systems in countries with different insurance models are recommended, in order to identify appropriate caring systems that meet the health needs of both migrants and the expectations of health staff. In summary, the provision of culturally sensitive care is very complex. Policy makers should be aware that the challenges of providing care to cross-border migrants cannot be overcome unless a conducive environment and sufficient institutional capacity are put in place.

### Ethics approval

This study has been approved by the ethical committee, the Institute for Development of Human Research Protection, Thailand (letter no. IHRP: 1778/2014)

## Additional files

Additional file 1: **Characteristics of the selected articles.** (DOCX 94 kb)
Additional file 2: **Key findings and quality assessment issues of the selected articles.** (DOCX 109 kb)

## References

[1] Internation Organization for Migration. World migration report 2010. The future of migration: Building capacities for change. Geneva: IOM; 2010.

[2] World Health Organization. The world health report 2006: working together for health. Geneva: WHO; 2006.

[3] Rausa B, Lloys LS, Loue S, Sajatovic M (2012). Immigration in the Global Context. Encyclopedia of immigrant health.

[4] Karl-Trummer U, Novak-Zezula S, Metzler B (2010). Access to health care for undocumented migrants in the EU: A first landscape of NowHereland. Eurohealth.

[5] Biswas D, Kristiansen M, Krasnik A, Norredam M (2011). Access to healthcare and alternative health-seeking strategies among undocumented migrants in Denmark. BMC Public Health.

[6] Macpherson DW, Gushulak BD, Macdonald L (2007). Health and foreign policy: influences of migration and population mobility. Bull World Health Organ.

[7] United Nations Programme on HIV/AIDS (2009). Background paper : People on the move – forced displacement and migrant populations. 24th Programme Coordinating Board – Thematic segment.

[8] World Health Organization. Public Health Aspects of Migration in Europe. Migration and health at the 2014 European Public Health (EPH) Conference 2015 [cited 12 July 2015]; Available from: http://www.euro.who.int/__data/assets/pdf_file/0010/269452/Public-Health-and-Migration-Newsletter-4th-Issue_NEWS_220115.pdf?ua=1

[9] World Health Organization: 60th Assembly (2007). Resolution WHA60.26 - Workers’ health: global plan of action. Resolution WHA6026.

[10] World Health Organization: 61st Assembly (2008). Resolution WHA61.17 - Health of migrants.

[11] Ullmann SH, Goldman N, Massey DS (2011). Healthier before they migrate, less healthy when they return? The health of returned migrants in Mexico. Soc Sci Med.

[12] Lassetter JH, Callister LC (2009). The impact of migration on the health of voluntary migrants in western societies. J Transcult Nurs.

[13] Spallek J, Zeeb H, Razum O (2011). What do we have to know from migrants’ past exposures to understand their health status? a life course approach. Emerg Themes Epidemiol.

[14] Malmusi D, Borrell C, Benach J (2010). Migration-related health inequalities: showing the complex interactions between gender, social class and place of origin. Soc Sci Med.

[15] De Maio F (2010). Immigration as pathogenic: a systematic review of the health of immigrants to Canada. Int J Equity Health.

[16] Almeida LM, Caldas J, Ayres-de-Campos D, Salcedo-Barrientos D, Dias S (2013). Maternal healthcare in migrants: a systematic review. Matern Child Health J.

[17] Brewin P, Jones A, Kelly M, McDonald M, Beasley E, Sturdy P, Bothamley G, Griffiths C (2006). Is screening for tuberculosis acceptable to immigrants? A qualitative study. Journal of Public Health.

[18] World Health Organization. The world health report 2006: working together for health. WHO; 2006.

[19] Ferlie EB, Shortell SM (2001). Improving the quality of health care in the United Kingdom and the United States: a framework for change. Milbank Q.

[20] Spencer L, Ritchie J, Lewis J, Dillon L (2003). Quality in Qualitative Evaluation: A framework for assessing research evidence.

[21] CASP. 10 Questions to make sense of qualitative research. 2013 [cited 1 June 2015]; Available from: http://media.wix.com/ugd/dded87_29c5b002d99342f788c6ac670e49f274.pdf

[22] Grewal SK, Bhagat R, Balneaves LG (2008). Perinatal beliefs and practices of immigrant Punjabi women living in Canada. JOGNN J Obstet Gynecol Neonatal Nurs.

[23] Thomas J, Harden A (2008). Methods for the thematic synthesis of qualitative research in systematic reviews. BMC Med Res Methodol.

[24] Britten N, Campbell R, Pope C, Donovan J, Morgan M, Pill R (2002). Using meta ethnography to synthesise qualitative research: a worked example. J Health Serv Res Policy.

[25] Abbott S, Riga M (2007). Delivering services to the Bangladeshi community: the views of healthcare professionals in East London. Public Health.

[26] Akhavan S (2012). Midwives views on factors that contribute to health care inequalities among immigrants in Sweden: a qualitative study. Int J Equity Health.

[27] Boerleider AW, Francke AL, van de Reep M, Mannien J, Wiegers TA, Deville WL (2014). “Being flexible and creative”: a qualitative study on maternity care assistants’ experiences with non-Western immigrant women. PLoS One.

[28] Briones-Vozmediano E, Goicolea I, Ortiz-Barreda GM, Gil-González D, Vives-Cases C (2014). Professionals’ perceptions of support resources for battered immigrant women: chronicle of an anticipated failure. J Interpers Violence.

[29] Byrskog U, Olsson P, Essen B, Allvin MK (2015). Being a bridge: Swedish antenatal care midwives’ encounters with Somali-born women and questions of violence; a qualitative study. BMC Pregnancy and Childbirth.

[30] Cross WM, Bloomer MJ (2010). Extending boundaries: clinical communication with culturally and linguistically diverse mental health clients and carers. Int J Ment Health Nurs.

[31] Dauvrin M, Lorant V, Sandhu S, Deville W, Dia H, Dias S, Gaddini A, Ioannidis E, Jensen NK, Kluge U (2012). Health care for irregular migrants: pragmatism across Europe: a qualitative study. BMC Res Notes.

[32] Donnelly TT, McKellin W (2007). Keeping healthy! Whose responsibility is it anyway? Vietnamese Canadian women and their healthcare providers’ perspectives. Nurs Inq.

[33] Eklof N, Hupli M, Leino-Kilpi H (2015). Nurses’ perceptions of working with immigrant patients and interpreters in Finland. Public Health Nurs.

[34] Englund ACD, Rydström I (2012). “I have to turn myself inside out”: Caring for immigrant families of children with asthma. Clin Nurs Res.

[35] Farley R, Askew D, Kay M (2014). Caring for refugees in general practice: Perspectives from the coalface. Australian Journal of Primary Health.

[36] Foley EE (2005). HIV/AIDS and African immigrant women in Philadelphia: Structural and cultural barriers to care. AIDS Care Psychol Socio-Med Asp AIDS HIV.

[37] Fowler N, Redwood-Campbell L, Molinaro E, Howard M, Kaczorowski J, Jafarpour M, Robinson S (2005). The 1999 international emergency humanitarian evacuation of the Kosovars to Canada: A qualitative study of service providers’ perspectives at the international, national and local levels. Int J Equity Health.

[38] Goldade K, Okuyemi KS (2012). Deservingness to state health services for South-South migrants: a preliminary study of Costa Rican providers’ views. Soc Sci Med.

[39] Hakonsen H, Lees K, Toverud EL (2014). Cultural barriers encountered by Norwegian community pharmacists in providing service to non-Western immigrant patients. Int J Clin Pharm.

[40] Health Protection Agency (2010). Understanding the health needs of migrants in the South East region: A Report by the South East Migrant Health Study Group on behalf of the Department of Health.

[41] Høye S, Severinsson E (2008). Intensive care nurses’ encounters with multicultural families in Norway: An exploratory study. Intensive Crit Care Nurs.

[42] Hultsjö S, Hjelm K (2005). Immigrants in emergency care: Swedish health care staff’s experiences. Int Nurs Rev.

[43] Kurth E, Jaeger FN, Zemp E, Tschudin S, Bischoff A (2010). Reproductive health care for asylum-seeking women - A challenge for health professionals. BMC Public Health.

[44] Lindsay S, King G, Klassen AF, Esses V, Stachel M (2012). Working with immigrant families raising a child with a disability: challenges and recommendations for healthcare and community service providers. Disabil Rehabil.

[45] Lyberg A, Viken B, Haruna M, Severinsson E (2012). Diversity and challenges in the management of maternity care for migrant women. J Nurs Manag.

[46] Manirankunda L, Loos J, Debackaere P, Nöstlinger C (2012). “It is not easy”: Challenges for provider-initiated HIV testing and counseling in Flanders, Belgium. AIDS Educ Prev.

[47] Munro K, Jarvis C, Kong LY, D’Souza V, Graves L (2013). Perspectives of family physicians on the care of uninsured pregnant women. J Obstet Gynaecol Can.

[48] Nicholas DB, Hendson L, Reis MD (2014). Connection versus disconnection: examining culturally competent care in the neonatal intensive care unit. Soc Work Health Care.

[49] O’Mahony JM, Donnelly TT (2007). The influence of culture on immigrant women’s mental health care experiences from the perspectives of health care providers. Issues Ment Health Nurs.

[50] Otero-Garcia L, Goicolea I, Gea-Sánchez M, Sanz-Barbero B (2013). Access to and use of sexual and reproductive health services provided by midwives among rural immigrant women in Spain: midwives’ perspectives. Glob Health Action.

[51] Pergert P, Ekblad S, Enskär K, Björk O (2008). Bridging obstacles to transcultural caring relationships-Tools discovered through interviews with staff in pediatric oncology care. Eur J Oncol Nurs.

[52] Rosenberg E, Richard C, Lussier MT, Abdool SN (2006). Intercultural communication competence in family medicine: lessons from the field. Patient Educ Couns.

[53] Samarasinghe K, Fridlund B, Arvidsson B (2010). Primary health care nurses’ promotion of involuntary migrant families’ health. Int Nurs Rev.

[54] Sandhu S, Bjerre NV, Dauvrin M, Dias S, Gaddini A, Greacen T, Ioannidis E, Kluge U, Jensen NK, Lamkaddem M (2013). Experiences with treating immigrants: a qualitative study in mental health services across 16 European countries. Soc Psychiatry Psychiatr Epidemiol.

[55] Suurmond J, Rupp I, Seeleman C, Goosen S, Stronks K (2013). The first contacts between healthcare providers and newly-arrived asylum seekers: A qualitative study about which issues need to be addressed. Public Health.

[56] Straßmayr C, Matanov A, Priebe S, Barros H, Canavan R, Díaz-Olalla JM, Gabor E, Gaddini A, Greacen T, Holcnerová P (2012). Mental health care for irregular migrants in Europe: Barriers and how they are overcome. BMC Public Health.

[57] Terraza-Núñez R, Vázquez LM, Vargas I, Lizana T (2011). Health professional perceptions regarding healthcare provision to immigrants in Catalonia. Int J Public Health.

[58] van den Ameele S, Keygnaert I, Rachidi A, Roelens K, Temmerman M (2013). The role of the healthcare sector in the prevention of sexual violence against sub-Saharan transmigrants in Morocco: a study of knowledge, attitudes and practices of healthcare workers. BMC Health Serv Res.

[59] Vangen S, Johansen REB, Sundby J, Træen B, Stray-Pedersen B (2004). Qualitative study of perinatal care experiences among Somali women and local health care professionals in Norway. Eur J Obstet Gynecol Reprod Biol.

[60] Wachtler C, Brorsson A, Troein M (2006). Meeting and treating cultural difference in primary care: a qualitative interview study. Fam Pract.

[61] Worth A, Irshad T, Bhopal R, Brown D, Lawton J, Grant E, Murray S, Kendall M, Adam J, Gardee R (2009). Vulnerability and access to care for South Asian Sikh and Muslim patients with life limiting illness in Scotland: prospective longitudinal qualitative study. BMJ.

[62] Isaacs S, Valaitis R, Newbold KB, Black M, Sargeant J (2013). Competence trust among providers as fundamental to a culturally competent primary healthcare system for immigrant families. Prim Health Care Res Dev.

[63] Kim-Godwin YS, Alexander JW, Felton G, Mackey MC, Kasakoff A (2006). Prerequisites to providing culturally competent care to Mexican migrant farmworkers: a Delphi study. J Cult Divers.

[64] Bischoff A, Hudelson P (2010). Access to healthcare interpreter services: where are we and where do we need to go?. Int J Environ Res Public Health.

[65] Ngo-Metzger Q, Massagli MP, Clarridge BR, Manocchia M, Davis RB, Iezzoni LI, Phillips RS (2003). Linguistic and cultural barriers to care. J Gen Intern Med.

[66] Binder P, Johnsdotter S, Essén B (2012). Conceptualising the prevention of adverse obstetric outcomes among immigrants using the ‘three delays’ framework in a high-income context. Soc Sci Med.

[67] Gray B, Hilder J, Donaldson H (2011). Why do we not use trained interpreters for all patients with limited English proficiency? Is there a place for using family members?. Australian Journal of Primary Health.

[68] Karamitri I, Bellali T, Galanis P, Kaitelidou D (2013). The accessibility of vulnerable groups to health services in Greece: a Delphi study on the perceptions of health professionals. Int J Health Plann Manag.

[69] Meershoek A, Krumeich A, Vos R (2011). The construction of ethnic differences in work incapacity risks: analysing ordering practices of physicians in the Netherlands. Soc Sci Med.

[70] BBC News. Should migrants have compulsory health checks? 2003 [cited 22 July 2014]; Available from: http://news.bbc.co.uk/1/hi/talking_point/3122199.stm

[71] Bracanovic T (2013). Against culturally sensitive bioethics. Med Health Care and Philos.

[72] Swendson C, Windsor C (1996). Rethinking cultural sensitivity. Nurs Inq.

[73] Gray BH, van Ginneken E (2012). Health care for undocumented migrants: European approaches. Issue Brief (Common Fund).

[74] United Nations, United Nations (1948). Universal Declaration of Human Rights. 217 A (III).

[75] Paisanpanichkul D (2008). Chapter 4: ‘Insurers’ of health insurance under domestic legal mandates in Thailand, UK and France, and international legal instruments. ‘Policy recommendations for developing health insurance for stateless/nationalityless population in Thailand’.

[76] Marrow HB (2012). Deserving to a point: unauthorized immigrants in San Francisco’s universal access healthcare model. Soc Sci Med.

[77] Mn R-B, Díez-Cornell M, Llorente JM (2013). Health-care access for migrants in Europe: the case of Spain. Lancet.

[78] Vanthuyne K, Meloni F, Ruiz-Casares M, Rousseau C, Ricard-Guay A (2013). Health workers’ perceptions of access to care for children and pregnant women with precarious immigration status: Health as a right or a privilege?. Soc Sci Med.

[79] Miklavcic A (2011). Canada’s non-status immigrants: negotiating access to health care and citizenship. Med Anthropol.

[80] Priebe S, Bogic M, Adany R, Bjerre NV, Dauvrin M, Devillé W, Dias S, Gaddini A, Greacen T, Kluge U, Rechel B, Mladovsky P, Devillé W, Rijks B, Petrova-Benedict R, McKee M (2011). Good practice in emergency care: views from practitioners. Migration and health in the European Union.

[81] Jensen NK, Norredam M, Draebel T, Bogic M, Priebe S, Krasnik A (2011). Providing medical care for undocumented migrants in Denmark: what are the challenges for health professionals?. BMC Health Serv Res.

[82] Ruiz-Casares M, Rousseau C, Laurin-Lamothe A, Rummens JA, Zelkowitz P, Crépeau F, Steinmetz N (2013). Access to health care for undocumented migrant children and pregnant women: The paradox between values and attitudes of health care professionals. Matern Child Health J.

[83] Hargreaves S, Holmes AH, Saxena S, Le Feuvre P, Farah W, Shafi G, Chaudry J, Khan H, Friedland JS (2008). Charging systems for migrants in primary care: the experiences of family doctors in a high-migrant area of London. Journal of Travel Medicine.

[84] De Genova N (2004). The legal production of Mexican/migrant ‘illegality’. Latino Studies.

[85] Van Der Leun J (2006). Excluding illegal migrants in The Netherlands: between national policies and local implementation. West European Politics.

[86] The immigration news. UK’s Strict Immigration Laws will Hurt Economy. 2014 [cited 23 July 2014]; Available from: http://news.visato.com/england/uks-strict-immigration-laws-will-hurt-economy/20120730/

[87] Lyons SM, O’Keeffe FM, Clarke AT, Staines A (2008). Cultural diversity in the Dublin maternity services: the experiences of maternity service providers when caring for ethnic minority women. Ethn Health.

[88] Gough D, Thomas J, Oliver S (2012). Clarifying differences between review designs and methods. Syst Rev.

[89] Voils CI, Sandelowski M, Barroso J, Hasselblad V (2008). Making sense of qualitative and quantitative findings in mixed research synthesis studies. Field Methods.

[90] Atkins S, Lewin S, Smith H, Engel M, Fretheim A, Volmink J (2008). Conducting a meta-ethnography of qualitative literature: lessons learnt. BMC Med Res Methodol.

[91] Bilger V, Hollomey C (2011). Policies on health care for undocumented migrants in EU27, country report: Switzerland.

[92] Walker PF, Barnett ED, Walker PF, Barnett ED (2007). An introduction to the field of refugee and immigrant healthcare. Immigrant Medicine.

