# Supplementary material for: Challenges in the provision of healthcare services for migrants: a systematic review through providers’ lens
Source: BMC Health Serv Res. 2015 Sep 17;15:390. doi: 10.1186/s12913-015-1065-z (PMC4574510; doi:10.1186/s12913-015-1065-z)
Supplement: Additional file 1: — Characteristics of the selected articles. (DOCX 94 kb) [file 12913_2015_1065_MOESM1_ESM.docx]

Additional file 1: Table S1: Characteristics of the selected articles

| Selected articles (author(s), year) | Country of study | Migrants' profiles | Type of services | Healthcare providers | Data collection techniques |
| --- | --- | --- | --- | --- | --- |
| 1. Abbot and Riga (2007) [[25](#_ENREF_25)] | UK | Bangladeshi community | Primary care | General practitioners (GPs), child development team, school nurses, etc | Focus group discussions (FGDs) |
| 1. Akhavan (2012) [[26](#_ENREF_26)] | Sweden | Immigrants, including undocumented persons and asylum seekers (mostly from Eastern Europe) | Primary care | Midwives | Semi-structured interviews |
| 1. Boerleider et al (2014) [[27](#_ENREF_27)] | Netherlands | Non-western immigrants (mostly from Turkey, Morocco, etc) | Postnatal care | Maternity care assistants | In-depth interviews (IDIs) |
| 1. Briones-Vozmediano et al (2014) [[28](#_ENREF_28)] | Spain | Battered immigrant women | Care for battered immigrant women | Social workers, psychologists, intercultural mediators, etc, who worked in non-profit organisations | IDIs and FGDs |
| 1. Byrskog et al (2015) [[29](#_ENREF_29)] | Sweden | Somali-born women | Antenatal care | Midwives | IDIs |
| 1. Cross and Bloomer (2010) [[30](#_ENREF_30)] | Australia | Migrant communities (nationality not specified) | Mental health care | Mental health clinicians | FGDs |
| 1. Dauvrin et al (2012) [[31](#_ENREF_31)] | 16 countries in the EUGATE project^*^ | Irregular migrants | Mental health services, Accident & Emergency (A&E) departments, and primary care | Clinicians and health managers | Semi-structured interviews |
| 1. Donnelly and McKellin (2007) [[32](#_ENREF_32)] | Canada | Vietnamese immigrants | Breast cancer and cervical cancer screening | Community physicians and nurses | IDIs |
| 1. Eklof et al (2015) [[33](#_ENREF_33)] | Finland | Migrants and asylum seekers (countries not specified) | Primary care | Community nurses | IDIs |
| 1. Englund and Rydstrom (2012) [[34](#_ENREF_34)] | Sweden | Non-western immigrant parents of children with asthma | Care for asthma patients | Nurses and physicians in asthma clinic | IDIS |
| 1. Farley et al (2014) [[35](#_ENREF_35)] | Australia | Refugees | Primary care | General practitioners (GPs), nurses and administrative staff | Semi-structured interviews and FGDs |
| 1. Foley (2005) [[36](#_ENREF_36)] | USA | HIV-positive African immigrants | HIV care | Medical practitioners and social workers | IDIs, informal interviews, and FGDs |
| 1. Fowler et al (2005) [[37](#_ENREF_37)] | Canada | Kosovar refugees | General health services | Family physicians, nurses, dentists, etc | IDIs |
| 1. Goldabe and Okuyemi (2011) [[38](#_ENREF_38)] | Costa Rica | Undocumented Nicaraguan migrants | 1. antenatal care 2. acute-fatal infectious diseases 3. sexually-transmitted diseases 4. occupational injuries | Nurses, physicians, and social workers | IDIs |
| 1. Hakonsen et al (2014) [[39](#_ENREF_39)] | Norway | Non-western immigrants | Community care | Community pharmacists | FGDs |
| 1. Health Protection Agency (2010) [[40](#_ENREF_40)] | UK | Refugees, asylum seekers, irregular migrants, undocumented people, over-stayers and family joiners | Various settings (eg, Primary Care Trust—PCT, local authority, and mental health trusts) | Health workers, type unspecified | Phone interview |
| 1. Hoye and Severinsson (2008) [[41](#_ENREF_41)] | Norway | Non-western immigrants | Critical illness care | Intensive care unit nurses | FGDs |
| 1. Hultsjo and Hjelm (2005) [[42](#_ENREF_42)] | Sweden | Refugees and asylum seekers | Emergency care, ambulatory care and intensive care | Nurses and assistant nurses | FGDs |
| 1. Kurth et al (2010) [[43](#_ENREF_43)] | Switzerland | Asylum seeking women | Delivery and maternity care | Physicians, nurses, midwives, psychologists and interpreters | Semi-structured interviews |
| 1. Lindsay et al (2012) [[44](#_ENREF_44)] | Canada | Immigrant families | Rehabilitation | Physiotherapists and social workers | IDIs and FGDs |
| 1. Lyberg et al (2012) [[45](#_ENREF_45)] | Norway | Female migrants with diverse ethnicities | Maternity care | Midwives and public health nurse | FGDs |
| 1. Manirankunda et al (2012) [[46](#_ENREF_46)] | Belgium | Sub-Saharan African migrants (SAMs) | HIV clinics | Nurses, midwives, and obstetricians | IDIs |
| 1. Munro et al (2013) [[47](#_ENREF_47)] | Canada | Uninsured pregnant women with precarious immigration status | Antenatal care and delivery | Family physicians | Semi-structured interviews |
| 1. Nicholas et al (2014) [[48](#_ENREF_48)] | Canada | Immigrant families, mostly from Asia and the Pacific region | Neonatal intensive care units | Neonatologists, nurse practitioners, social workers, administrative staff, etc | FGDs |
| 1. O'mahony and Donnelly (2007) [[49](#_ENREF_49)] | Canada | Immigrant women | Mental health care | Social workers, physicians and nurses | IDIs |
| 1. Otero-Garcia et al (2013) [[50](#_ENREF_50)] | Spain | Immigrant women | Sexual and reproductive health services | Midwives in primary care | IDIs |
| 1. Pergert et al (2008) [[51](#_ENREF_51)] | Sweden | Immigrant patients in paediatric oncology units | Care for children with cancer | Nurses and nurse aides | FGDs and IDIs |
| 1. Rosenberg et al (2006) [[52](#_ENREF_52)] | Canada | Immigrant patients, mostly from Asia and Africa | Primary care | Family physicians | Non participant observations and interviews |
| 1. Samarasinghe et al (2010) [[53](#_ENREF_53)] | Sweden | Involuntary migrants | Nurse-led primary care clinics | Primary health care nurses (PCHNs) | Semi-structured interviews |
| 1. Sandu et al (2013) [[54](#_ENREF_54)] | 16 countries in the EUGATE project^*^ | First-generation immigrants born outside the country of current residence (including regular immigrants, irregular immigrants, asylum seekers, refugees and human trafficking victims) | Mental health care | Psychiatrists, mental health nurses, social workers, etc | Semi-structured interviews |
| 1. Straßmayr et al (2012) [[56](#_ENREF_56)] | 14 European countries^#^ | Irregular migrants | Mental health care | Mental health care experts | Semi-structured interviews |
| 1. Suurmond et al (2013) [[55](#_ENREF_55)] | Netherlands | Asylum seekers | Primary care (first contact care) | Physicians and nurse practitioners | Group interviews |
| 1. Terraza-Nu´n˜ez et al (2011) [[57](#_ENREF_57)] | Spain | Immigrant populations, mostly from Morocco, Romania, and Latin America countries | Primary and secondary care | Health managers and health professionals in primary and secondary care | Semi-structured interviews |
| 1. van den Ameele et al (2013) [[58](#_ENREF_58)] | Morocco | Sub-Saharan migrants | Sexual violence prevention | General practitioners, community workers, and obstetricians | Semi-structured interviews |
| 1. Vangen et al (2004) [[59](#_ENREF_59)] | Norway | Immigrant pregnant women from Somalia | Antenatal and delivery care | Gynaecologists, nurses and midwives | IDIs |
| 1. Wachtler et al (2006) [[60](#_ENREF_60)] | Sweden | Immigrant population, countries of origin not specified | Primary care | GPs | Semi-structured interviews |
| 1. Worth et al (2009) [[61](#_ENREF_61)] | UK | Asian Sikh and Muslim migrant communities | Hospitals, hospices, home care: life limiting illness (end-of-life care) | GPs, specialist nurses, social workers, and hospital manager | IDIs |

Note: * Austria, Belgium, Denmark, Finland, France, Italy, Lithuania, Germany, Greece, Hungary, the Netherlands, Poland, Portugal, Spain, Sweden, and the United Kingdom

# Austria, Belgium, Czech Republic, France, Germany, Hungary, Ireland, Italy, the Netherlands, Poland, Portugal, Spain, Sweden, and United Kingdom
