# Supplementary material for: Challenges in the provision of healthcare services for migrants: a systematic review through providers’ lens
Source: BMC Health Serv Res. 2015 Sep 17;15:390. doi: 10.1186/s12913-015-1065-z (PMC4574510; doi:10.1186/s12913-015-1065-z)
Supplement: Additional file 2: — Key findings and quality assessment issues of the selected articles. (DOCX 109 kb) [file 12913_2015_1065_MOESM2_ESM.docx]

Additional file 2: Table S3: Key findings and quality assessment issues of the selected articles

| Selected articles (author(s), year) | Objective(s) or research question(s) | Interaction with migrant patients | Interaction with providers' workplace (infrastructure and/or resources) | Interaction with external environment (social values, laws, economics, and politics) |
| --- | --- | --- | --- | --- |
| 1. Abbot and Riga (2007) [[25](#_ENREF_25)] | To explore the views of primary care staff about delivering services to the local Bangladeshi community | Language and religious difference served as key difficulties in service provision | Not clearly described | Peer pressure from families and communities played important role in inhibiting or promoting health services. For example, Families were typically large and thus placed multiple demands on parents, who might decide that a healthcare appointment was not a priority |
| 1. Akhavan (2012) [[26](#_ENREF_26)] | To explore the views of midwives on the factors that contribute to health care inequality amongst immigrants | Midwives believed that health care inequality amongst immigrants migrant was the result of miscommunication, arising due to a shortage of meeting time, language barriers, and limited patient-caregiver trust. | Due to capacity/resources constraint in the workplace where these midwives were working, the time allotted for a consultative meeting with migrants was very limited. All participants agreed that having culturally diverse health staff was important in providing culturally sensitive care. Yet, some participants considered another way by stating that having subgroup-specific health services might lead to poorer quality of care because qualified health staff were likely refuse to work in such areas. | Patriarchal culture could create misunderstanding between midwives and migrant patients. |
| 1. Boerleider et al (2014) [[27](#_ENREF_27)] | (1) How do Dutch Maternal Care Assistants (MCAs) feel about providing care to non-western clients?; and (2) Do Dutch MCAs adjust their care to non-western clients and if so in what ways? | MCAs often found that migrant clients had limited knowledge in maternity care. Language difference also served as another barrier in health education. Some MCAs described caring non-Dutch speaking mothers intensive and sometimes frustrating. | Telephone professional interpreters were asked to help MCAs in communicating with non-western mothers. However, this service was not always available. | Family involvement played a pivotal role in maternity care. MCAs needed to put more effort in understanding cultural values of their clients. Unusual customs, such as a husband refusing to shake hands, could be perceived as an offence, but should be respected. |
| 1. Briones-Vozmediano et al (2014) [[28](#_ENREF_28)] | To explore the experience of service providers in Spain concerning their daily professional encountered with battered immigrant women | Some providers felt frustrated with the decision of immigrant women, particularly those living in low socioeconomic status, who abandoned the health seeking process due to ignorance of the system. | Some professionals attributed the abandonment of the help seeking process of a victim to the failure of the system and the ineffectiveness of existing resources (lodging an official complain but no solution). | Many immigrant women, especially those in Arab families, failed to escape the violence cycle due to the submission to patriarchal belief. |
| 1. Byrskog et al (2015) [[29](#_ENREF_29)] | To explore ways antenatal care midwives in Sweden work with Somali born women and the questions of exposure to violence. | Almost all midwife informants broached questions about violence exposure with Somali born women, but they rarely encountered ongoing violence. Limited communication and divergent life situations led to barriers in determining violence towards Somali born women. | Not clearly described | Some midwives experienced that in relation to the Somali born women, they lacked the background information vis-à-vis cultural and religious conceptions of health, family life, value systems, and violence, which might hinder the quality of care. |
| 1. Cross and Bloomer (2010) [[30](#_ENREF_30)] | (1) To explore how mental health clinicians modify their communication practice to address cultural differences and promote client self-disclosure; and (2) To identify the practical experiences that clinicians have used when interacting with people from culturally diverse groups | Participants in this study recognised language as one of the communication difficulties. Besides, gender role in migrant community was another issue that made patients adapted their approach. In cases of sexual assault, abuse and childhood trauma, female clinicians were preferred. | Not clearly described | Participants in this study tried to understand the belief systems of the many cultural groups they were caring. Some migrant communities still understood that mental illness was a punishment from god or superstition. |
| 1. Dauvrin et al (2012) [[31](#_ENREF_31)] | To investigate the experiences of health professionals in providing care to irregular migrants in three types of health care service (maternity care, accident & emergency care, and primary care) across 16 European countries^*^ | Health workers in accident and emergency (A&E) departments reported less of a difference between the care for migrant patients and for native patients in a regular situation than did respondents in primary care and mental health services. Concern over language differences was more apparent in mental health services than in A&E departments. | Professionals in primary care and mental health services felt more difficulties in performing further diagnostic and/or therapeutic interventions due to the restriction against health care access of irregular migrants. Some clinicians solved this nuisance by prescribing medicines for their own names if patients cannot afford the drug expense. | Even in countries with full rights of health care access for irregular migrants, there were still problems when referral was needed. Delay of treatment occurred frequently as providers and patients needed to wait until legal issue of the patients was resolved. Notifying police was a rare practice, even in countries where healthcare providers were obliged to do so. |
| 1. Donnelly and McKellin (2007) [[32](#_ENREF_32)] | To understand views of healthcare providers in catering breast and cervical cancer screening services for Vietnamese women in Candata | The majority of healthcare provider informants were aware that Vietnamese women were hesitant and embarrassed about breast and cervical examination due to cultural differences. | Healthcare providers adpate their routine pracitces to facilitate the service use of Vietnamese women by providing flexible hours of operation for the healthcare clinic, physicians reminding women of their check-ups, and having more accessible educational materials for women | The study suggested that the Canadian government paid little attention on the promotion of cancer screening and specifically for the Vietnamese migrants. This was identified as the most significant barrier to screening services. In some provinces, services for immigrants had suffered the biggest funding cut backs. |
| 1. Eklof et al (2015) [[33](#_ENREF_33)] | To describe nurses perceptions when using interpreters in primary health care nursing with migrant patients | Nurses were usually aware of the importance of interpreters in tackling language differences of their migrant clients. However, some nurses considered interpreters as translation machines and as cultural brokers. | The ordering and availability of interpreters seemed to be challenging and time-consuming. With references to some nurses’ experience, access to the interpreter service by phone was difficult and increased workload, specifically in urgent situations. | In Finland, there were several regulations regarding the use of interpreters. The interpreting costs in health  care were paid for by the government if the patient was a asylum seeker and being in an integration program; if not, the costs would be paid by a municipality, which usually had strict guidelines for the use of interpreters. This contributed to a number of hurdles in service provision. |
| 1. Englund and Rydstrom (2012) [[34](#_ENREF_34)] | To gain a broader insight of the challenges healthcare professionals faces in their encounters with non-western immigrant parents of children with asthma | Health professional informants perceived that language differences and unfamiliarity with the western medicine of immigrant families sometimes created frustration and conflicts between healthcare providers and their clients. | Not clearly described | A strong belief in some families that trusted male physicians far over female nurses could create uncomfortable feeling and sense of powerlessness in female nurses. Despite having telephone consultation service by nurses in Swedish healthcare system, many immigrant families only accepted personal contact with (male) physician instead of using nurse-led telephone advice. |
| 1. Farley et al (2014) [[35](#_ENREF_35)] | To explore enabling factors and barriers healthcare providers experienced in providing care to refugees | Participants described communication difficulties as one of the most important barriers to refugee health care. | Interpreter services were a crucial enabler of refugee health care but were also time consuming, often unavailable and sometimes of questionable reliability. | Not clearly described |
| 1. Foley (2005) [[36](#_ENREF_36)] | To examine perspectives of medical practitioners and social workers that were bestowing clinical care and support services to African immigrants living with HIV/AIDS | The provider informants expressed a keen awareness of the broad cultural gulf that secluded them from their African patients. The difficulty of communicating effectively with patients who had little or no formal education and limited English or French fluency was their greatest frustration. | Nurses and social workers at times adapted the routine service guideline of a facility in order to better suit expectation and cultural belief of African HIV patients. These strategies included giving African patients their medications in unlabelled bottles, delivery of medications to locations other than their patients’ homes, and helping women negotiate condom use with male partners without disclosing their HIV status. | In order to be insured at the city heath centres, patients must first provide proof of residence in the city of Philadelphia. Yet, African women often had no documentation in their own name because they lived with male partners or relatives. In spite of these difficulties, many case managers, nurses, and social workers found ways to assist both documented and undocumented patients without health insurance through several strategies, such as asking for fund from special government programmes or from other supporting agencies. |
| 1. Fowler et al (2005) [[37](#_ENREF_37)] | To investigate the main challenges and successes of the Kosovar arrival, from international, national, and local perspectives | Not clearly described | Many health professional respondents expressed concern over the location of medical records and the inability to access this information in a timely manner since the information system of a Canadian health facility was not integrated with that in the country of origin of the refugees. | The Canadian regulation allowed Kosovars to received medical care through the Interim Federal Health (IFH) programme. However, some services were not covered for free of charge (home health care, eye glasses for refractive error, etc). This confusion rendered difficulties on healthcare providers. |
| 1. Goldabe and Okuyemi (2011) [[38](#_ENREF_38)] | To explore attitudes of providers in Costa Rica concerning deservingness to care of Nicaraguan undocumented migrants | Not clearly described | Not clearly described | Normally, by law, undocumented migrants were barred against public health services with only 3 exceptions, namely, emergency services, health care for children and adolescent until age 18 years, and prenatal care. Providers, however, considered undocumented migrants should not deserve medical treatment for occupational injuries as profits did not benefit health of the national population but went to individual companies. |
| 1. Hakonsen et al (2014) [[39](#_ENREF_39)] | To determine the cultural barriers met by Norwegian community pharmacists in providing service to non-western immigrant patients and to outline how they were being addressed | The pharmacist participants found that the presence of language barrier made the service provision for non-western immigrants challenging, and they were uncomfortable with situations where family or friends acted as interpreters, especially children. | Not clearly described | The lack of public interpreter services in community  pharmacies was a dilemma since Norwegian regulations  did not allow unskilled persons to act as interpreters. This situation was contradicting the hospital service guideline where professional interpreters might be required. |
| 1. Health Protection Agency (2010) [[40](#_ENREF_40)] | To analyse the use of services in various types of migrants in UK and to investigate the needs of professionals working with migrant patients | Respondents expressed concerns about language barriers, which impeded the provision of effective services, in particular, mental health care. | Not clearly described | Respondents briefly described confusion in the National Health Service (NHS)'s regulation. The UK health professionals thus entrusted non-statutory organisations or civil networks to some services (eg, housing aid) which were not regularly available to certain migrant groups, eg, vulnerable adults, migrants' relatives and dependants. |
| 1. Hoye and Severinsson (2008) [[41](#_ENREF_41)] | To explore the perception of intensive care unit (ICU) nurses with regards to their encounters with families of culturally diverse patients | The ICU nurses became insecure when they were encountering cultural differences. | The multicultural families seemed to expanded, relative to Norwegian families. The situations that produced stress were characterised by rooms crowded with family members  and large numbers of visitors, which might hamper nursing procedures in the patient’s room. | ICU nurses felt that, due to patriarchal views held by immigrant families, female nurses often received lack of respect from the ethnic groups. |
| 1. Hultsjo and Hjelm (2005) [[42](#_ENREF_42)] | To identify if healthcare staff in somatic and psychiatric emergency care experienced any problems in the services for migrants | All respondents expressed serious concerns over language barrier and difficulty to address the traumatic experiences of migrants. | Difficulties in finding an interpreter, especially at night, and minority language, and shortage of healthcare staff were regarded as the main setback in service provision in all ward types. | Not clearly described |
| 1. Kurth et al (2010) [[43](#_ENREF_43)] | To explore the perceptions of healthcare professionals caring for asylum seeking women in the Women’s Clinic of the University Hospital in the city of Basel, Switzerland | Language barriers were identified as a major struggle for health professionals in providing care. Health care providers also met major emotional challenges when taking care of asylum seekers. | Not clearly described | The Swiss government attempted to reduce health expenditure by limiting the asylum seekers’ choice of where to seek care and assigning them to primary health care providers’ networks, so called, Health Maintenance Organisation (HMO). The problems, faced by physicians, were that they were often required to act in an official capacity on behalf of the authorities in charge of the asylum process, and they were also forced to make decisions in controlling expenditure to fulfill the requirements of the HMO. |
| 1. Lindsay et al (2012) [[44](#_ENREF_44)] | To obtain a better understanding of the experiences of service providers working with immigrant families raising children with physical disability | Healthcare and community service providers often encountered several challenges (such as language difficulties, unfamiliarity with Canadian health system of a patient, and discrepancy in the view concerning disability) in providing care to immigrant families. | Though professional interpreters were available, the use of professional interpreters was quite limited since it always added time on the clients' appointment. | Not clearly described |
| 1. Lyberg et al (2012) [[45](#_ENREF_45)] | To illuminate public health nurses' and midwives' perceptions of managing and supporting prenatal and postnatal migrant patients in Norway | Respondents experienced that general maternal care for Norwegian mothers did not match needs of female migrants. Posters and leaflets were at times too abstract. Linguistic and cultural barriers of migrants often shaped how providers delivered services. Some respondents considered videotape education instead of face-to-face meeting was more useful in keeping patients' privacy. | Respondents complained over the quality and availability of interpreting service in their workplaces. Male interpreters did not understand vocabularies commonly used in maternal care, and this could create distrust between providers and patients. | Not clearly described |
| 1. Manirankunda et al (2012) [[46](#_ENREF_46)] | To identify physicians’ HIV testing practices and barriers to managing provider-initiated HIV testing and counselling (PITC) for Sub-Saharan African migrants (SAM) in Belgium | Some health professionals were ignorant of the high prevalence of HIV epidemic amongst SAMs. Lack of expertise in discussing sexuality and lack of time also served as key barriers in implementing PITC. | Not clearly described | The issues of racism and shaky legal status of immigrants considerably affected the decision of doctors in undertaking PITC. Some doctors felt that carrying out an HIV test amongst undocumented migrants who might be deported at any time was unethical since they could not assure proper follow-up care. Some providers perceived promoting PITC for SAMs as a discriminatory practice, and might make them being fear of being accused of racism when suggesting an HIV test. |
| 1. Munro et al (2013) [[47](#_ENREF_47)] | To explore the perspectives of family physicians who provided services to uninsured pregnant women with precarious immigration status | Poverty and lack of understanding in the Canadian's insurance system played a major role in inhibiting access to care of immigrants. Nevertheless, care of uninsured women was generally thought to be a professional obligation, regardless of the woman’s ability to pay. | Logistically, physicians had difficulty accessing prenatal resources for their uninsured migrant patients. For example, social services were available only to officially recognised immigrants or refugees. | Many respondents altered standard of care in order to avoid cost incurred on uninsured immigrants, and to avoid referrals to specialist physicians. In Canada, refugees and refugee claimants were insured through the Interim Federal Health Programme. Quebec province imposed a 3-month delay on the acquisition of health insurance for newly arrived immigrants. However, due to administrative delays, some individuals with a right to public insurance found themselves without coverage. |
| 1. Nicholas et al (2014) [[48](#_ENREF_48)] | To examine cross-cultural care from the healthcare providers' perspective within two tertiary level Neonatal Intensive Care Units (NICUs) | Language and communication differences constantly presented barriers between families and providers. Healthcare providers participants felt that communication attempts were periodically impeded, reportedly heightened by the intensity and frequent shifting nature of information in the NICU. | Negligible staff representation in the ethnicities of families was noted. Limited availability of complementary and alternative treatment options was thought to limit capacity for cross-cultural care. There was a lack of cultural brokers and translators. | Conflicting cultural norms between families and healthcare providers were reported to impose inter-personal friction and feelings of being misunderstood and disrespected. Birthing rites and rituals were identified as culturally nuanced, yet often poorly understood and at times, disrespected and subjected to pejorative judgment. |
| 1. O'mahony and Donnelly (2007) [[49](#_ENREF_49)] | To examine concerns of healthcare provider in managing mental health care for immigrant women | The participants often mentioned that immigrant women’s misunderstandings of Western biomedicine and their unfamiliarity with mental health care service affected how these women sought help. Another difficulty raised was the immigrant women’s unfamiliarity with mental health services and lack of awareness in the availability of mental health support. | Not clearly described | All healthcare providers viewed the cultural and social stigma attached to mental illness as a key barrier to accessing mental health services for immigrant women. Some respondents expressed that in many cultures there were significant negative feelings towards mental illness and the taking of medication. |
| 1. Otero-Garcia et al (2013) [[50](#_ENREF_50)] | To explore the perceptions of midwives who provided maternity services, including sexual and reproductive programme, for immigrant women | Midwives explained that language and cultural differences, including gender inequity, were significant barriers to care. In some immigrant families, decision of men prevailed over women's decision (for example, barring women from family planning). | Not clearly described | Not clearly described |
| 1. Pergert et al (2008) [[51](#_ENREF_51)] | To gain knowledge about how health-care staff continuously resolve obstacles to trans-cultural caring relationships as they cared for immigrant families in paediatric oncology care units. | Nurses in paediatric oncology unit expressed their concern over linguistic difference. Several strategies, including non verbal communication using 'signs' and 'printed information', were used to bridge this obstacle. | The organisation had adapted its usual care policy by allocating extra time for immigrant patients, and striving for a diversity of healthcare staff in term of ethnic background. | Not clearly described |
| 1. Rosenberg et al (2006) [[52](#_ENREF_52)] | To explore challenges for immigrant patients and family physicians, involving in intercultural communication (ICC). | Some physicians reported that  when language barrier arose, they were more likely to  by-pass psychosocial aspects of the health problem since it was more time consuming than general somatic care. | Not clearly described | Physicians used incorrect beliefs about the expression of distress and illness experience in the patient’s culture. In some cases, physicians viewed patient’s behaviours as normal for a person of the given culture (such as tears and rotten words), while the same behaviour would be perceived as psychological distress in another culture. |
| 1. Samarasinghe et al (2010) [[53](#_ENREF_53)] | To describe the promotion of health in involuntary migrant families in cultural transition through the views of Swedish primary health care nurses (PHCNs) | Some PCHNs approached patients by focusing only on somatic health of individuals as they deemed expanding more than physical health was time-consuming, labour-intensive and sometimes costly due to translation fees. | Not clearly described | Some PHCNs empowered immigrant family members to be able to integrate into Swedish society by working closely with social workers and other healthcare workers, preschool teachers, etc. |
| 1. Sandu et al (2013) [[54](#_ENREF_54)] | To investigate professionals’ experiences of catering care to immigrants in districts densely populated with immigrants in 16 European countries^*^ | Interviewees noted a general concern with the development of trust and the particular need to establish it with immigrant patients who might be distrustful of authorities, or were unfamiliar with the health care system in a host country | Not clearly described | Divergent belief systems served as explanatory models for immigrant patients’ experiences of mental illness. These might hamper diagnosis and conflict with the practitioners’ understandings, resulting in differentiating between certain beliefs and symptoms. Usually, the challenge for practitioners was distinguishing what was a culturally normal response from what was an indication of mental pathology. |
| 1. Straßmayr et al (2012) [[56](#_ENREF_56)] | Objective 1—to identify barriers to mental health care in irregular migrants; Objective 2—to identify how health professionals tackle these problems in real practice | Respondents pointed out that decisions that should be made by clinicians were usually left to administrative staff with control over access to care. This in turn could result in irregular migrants being denied access due to arbitrariness and discrimination. | A general shortage of resources and limited capacities in mental health services were reported. Problems included long waiting lists; restricted availability of psychotherapy and psychological treatment. Physicians thus usually employed informal networks and non-government organisations to cover these unfilled gaps. | Experts from the countries that provided no legal access to mental health care for irregular migrants beyond emergency care described this lack of legal entitlement as the main barrier. In addition, a lack of awareness amongst care providers of what entitlements to health care are in place for migrants was reported as a significant barrier since it led to irregular migrants being left with a minimum of medical attendance despite having the right to receive care. |
| 1. Suurmond et al (2013) [[55](#_ENREF_55)] | To explore insight in the specific issues that healthcare providers were required to address in the first contacts with newly arrived asylum seekers. | The respondents felt that asylum seekers might have little knowledge about the way their body functions, and care providers might need to put a lot of effort in clarifying medical treatment. It was questionable to screen (mental) health problems when there was no guarantee for follow-up care. Thus, not all respondents were not strict to the screening guideline. | Not clearly described | Not clearly described |
| 1. Terraza-Nu´n˜ez et al (2011) [[57](#_ENREF_57)] | To analyse health personnel perceptions concerning the provision of care to immigrant population | Providing healthcare to immigrants created feelings of distress, overload and exhaustion in health professionals, especially in primary care level. Communication barriers emerged as one of the main problems. | Not clearly described | Informants ascribed the inadequacy of resources to an absence of suitable planning on the side of the health authority (Department of Health), as well as to its lethargy in adapting the health system (physical and human resources, regulations and clinical instruments) to the ‘‘sudden’’ increase in population size and needs of immigrants’ population. |
| 1. van den Ameele et al (2013) [[58](#_ENREF_58)] | To identify the current role and position of the healthcare sector in Morocco towards the prevention of sexual violence against sub-Saharan migrants | Respondents acknowledged the need for appropriate prevention of sexual violence against migrants, but differences in language, beliefs and cultures, occasionally, encumbered health care workers to tracking traumatic experiences and indentifying victim cases. | Limitations of the Moroccan public health sector re the response to sexual violence included Insufficient staffing, structures and resources. Accordingly, in practice, several informants indicated that  Trans-migrants relied entirely on help from NGOs. | Participants considered it was their responsibility to provide evidence of the violence and to refer immigrant victims to legal assistance. However, some providers viewed that reporting the presence of illegal migrants to police would increase risk of being deported, and such practice also contradicted the professional norm. |
| 1. Vangen et al (2004) [[59](#_ENREF_59)] | To explore how perinatal care practice might influence labour outcomes amongst circumcised women. | Health care professionals were uncertain about delivery procedures for infibulated women and occasionally caesarean sections were done in lieu of defibulation. Neglect of circumcision might lead to unnecessary caesarean sections or even adverse birth outcomes. | The communication between outpatient clinics and the hospitals re the management of infibulation was poor. The antenatal clinics had stopped referring women to the hospital for antenatal defibulation since their requests had been refused. | Not clearly described |
| 1. Wachtler et al (2006) [[60](#_ENREF_60)] | to examine how consultations with immigrant patients were understood by general practitioners (GPs) and how GPs managed these consultations | GPs conducted their consultations with immigrant patients in the same way that they conducted all their consultations. These culturally charged consultations were difficult, emotional, and led to a feeling of failure if the GPs felt they had no way of solving  the problem of failed communication, and therefore  could not be a good physician to their patients. | Not clearly described | Not clearly described |
| 1. Worth et al (2009) [[61](#_ENREF_61)] | To examine the experiences of South Asian Sikh and Muslim patients (and their families) in Scotland with life limiting illness and to identify how to overcome these problems | Most healthcare and social care professionals expressed intentions to provide equitable care both migrants and normal citizens, but their aim was hampered by language difficulties and lack of understanding of Muslim culture/tradition. The situation was more complicated when engaging with life limited illness, where bereavement, death and dying came into play since emotional troubles were difficult to address openly. | Health care services often faced difficulty in managing basic needs under Muslim culture, such as, the Halal diet, and need for specific hygiene practices, such as Wudu (ritual ablution preceding daily prayers), which were not usually prepared in the routine practice. | Not clearly described |

Note: * Austria, Belgium, Denmark, Finland, France, Italy, Lithuania, Germany, Greece, Hungary, the Netherlands, Poland, Portugal, Spain, Sweden, and the United Kingdom
